# Development and evaluation of RhizoQOL, a quality-of-life caregiver-reported survey for rhizomelic chondrodysplasia punctata, a rare peroxisomal disorder

**DOI:** 10.1186/s13023-025-03660-0

**Published:** 2025-03-31

**Authors:** Mousumi Bose, Tahra C. Anglade, Chelsea I. Donlon, Adrian L. Kerrihard, Hila F. Berger, Ariel S. Berkowitz, Shawn A. Ritchie, Tara M. Smith

**Affiliations:** 1 Department of Nutrition and Food Studies, College for Community Health, Montclair State University, 1 Normal Avenue, UN 2154, Montclair, NJ 07043 USA; 2 Department of Psychology, College of Arts and Sciences, Howard University, 2400 Sixth Street NW, Washington, DC 20059 USA; 3 Office for Research, Rutgers University, 33 Knightsbridge Road, 2nd Floor East, Piscataway, NJ 08854 USA; 4 Med-Life Discoveries LP, Saskatoon, SK S7N2X8 Canada

**Keywords:** Rare disease, Rhizomelic chondrodysplasia punctata, Quality of life, Caregiver, Survey, Caregiver report, Global impression of severity, Focus groups, Cognitive interviewing, Psychometrics

## Abstract

**Background:**

Rhizomelic chondrodysplasia punctata (RCDP) is a rare genetic disorder characterized by symptoms such as respiratory dysfunction, seizures, orthopedic issues, and neurodevelopmental delay. Potential therapeutics for RCDP warrant the development of clinical outcome assessments to assess the efficacy of treatment and the well-being of patients. Our study aimed to develop a valid quality-of-life (QOL) caregiver-reported survey instrument, RhizoQOL, to be used as a supportive endpoint in RCDP clinical trials.

**Methods:**

Development of the RhizoQOL survey tool included three RCDP caregiver focus groups to elicit concepts to serve as potential domains in a QOL survey instrument for RCDP, pilot survey development and initial testing, cognitive interviewing of revised survey drafts to determine content validity, as well as a three-month longitudinal study for reliability and internal consistency of the survey instrument.

**Results:**

Twenty-eight caregivers participated in the focus groups, reporting that concepts that could be appropriate domains of QOL in RCDP include psychosocial behavior, feeding symptoms, mobility symptoms, respiratory symptoms, seizures and related activity, and impact of treatment. Following pilot survey testing (*n* = 22) and stakeholder feedback, a revised pilot survey instrument was administered to five caregivers for cognitive interviewing. This resulted in a revised survey instrument with 31 question items, six domains, and a 1–5 Likert scale item response assessing frequency or severity of event in the question item. Longitudinal testing (*n* = 18) of the revised survey instrument found the average response score was 1.98 ± 0.97 for all question items, and a Cronbach’s alpha value of 0.856, suggesting strong intra-survey question reliability. Using individual question item results from reliability testing, linear regression modeling, and testing for required magnitude of significant treatment effects, eight question items were removed from the survey instrument, resulting in a total of 23 question items within 6 discrete domains.

**Conclusions:**

The final RhizoQOL survey instrument, consisting of 23 questions, assesses the symptoms and experiences of RCDP patients as observed by caregivers and serves as a novel clinical outcome assessment for RCDP therapeutic clinical trials to assess the impacts of RCDP and support the overall effectiveness of treatments.

**Supplementary Information:**

The online version contains supplementary material available at 10.1186/s13023-025-03660-0.

## Background

Rhizomelic chondrodysplasia punctata (RCDP) is an ultra-rare genetic disorder (estimated prevalence 1 per 100,000 individuals) caused by impaired function in one of the proteins in the plasmalogen biosynthesis pathway within the peroxisomes of eukaryotic cells [1]. Plasmalogens are membrane phospholipids that are defined by the presence of a vinyl-ether bond at the *sn*1 position of the glycerol backbone. Five subtypes of RCDP (RCDP1-5) have been reported, each caused by a mutation in a separate gene. The most common form is RCDP1 caused by pathogenic variants in the *PEX7* gene [2], which encodes the PEX7 receptor, responsible for importing alkylglycerone phosphate synthase (AGPS) into the peroxisome. Three subtypes are caused by mutations in genes encoding peroxisomal enzymes at the beginning of the plasmalogen biosynthesis pathway glycerophosphate-O-acyltransferase (*GNPAT*, RCDP2), (*AGPS*, RCDP3) [3], and fatty alcohol reductase 1 (*FAR1*, RCDP4) [4]. Additionally, RCDP5 results from a specific mutation in the *PEX5* gene that causes pathogenic changes in the long isoform of the PEX5 protein, impairing its ability to recognize the PEX7 receptor, thereby preventing its targeting to the peroxisome [5]. The consequence of any of these mutations is a severely impaired capacity to synthesize plasmalogens, with disease severity correlating closely with residual plasmalogen levels [6]. While genetic testing is commonly used to confirm diagnosis, RCDP can be diagnosed clinically through radiological findings combined with biochemical confirmation of reduced plasmalogen but normal levels of very long fatty acids (VLCFA) concentrations in red blood cells [7].

Clinically, RCDP is quite heterogeneous but often presents with shortening of the humerus and femur (rhizomelia), punctate calcification in cartilage (chondrodysplasia punctata), joint contractures, cataracts, and significant neurodevelopmental delay [8], as well as cervical spine deformities (e.g., spinal stenosis) [9]. Other symptoms may include respiratory dysfunction and seizures [10] and associated orthopedic issues caused by contractures [7].

Currently, treatment for RCDP is exclusively symptomatic and supportive [7]. Nevertheless, potential therapeutic targets for RCDP have been identified [11, 12], which aim to address the underlying cause of disease, plasmalogen deficiency, and will hopefully allow for better clinical management of RCDP compared to current supportive approaches.

The clinical heterogeneity, as well as the limited and largely outdated published natural history of RCDP, presents challenges in the identification of appropriate endpoints for clinical trials. The need to characterize disease presentation, natural history, and viable clinical endpoints is a common challenge encountered within the rare disease drug development space. Rare disease stakeholders from multiple sectors have emphasized the importance of developing clinical endpoints that are more representative of the disease and therefore more meaningful to patients [13]. In an effort to prioritize these outcomes there is increasing interest in the development of clinical outcome assessments such as patient- and observer-reported quality-of-life (QOL) survey instruments which aim to capture the overall well-being, functioning, effectiveness of therapeutic options, and overall healthcare needs of patients. The Food and Drug Administration (FDA) and other expert task forces have provided guidelines regarding the development, validation of content, and effective use of validated patient- and observer-reported outcome instruments for health-related concepts such as quality-of-life in clinical trials [14, 15]. The development of disease-specific clinical outcome assessments has been demonstrated to be useful within rare disease patient populations, with newly developed disease-specific clinical outcome assessments employed as endpoints in clinical trials [16].

Currently, there are no validated clinical outcome assessments specific to RCDP, presenting an obstacle in bringing therapeutics for RCDP to market. Moreover, our extensive research found no existing survey assessment tools consisting of measurement outcomes or domains relevant or appropriate to RCDP. The broad goal of this study was to establish a foundation for the development of a valid QOL survey instrument for RCDP which could be included as an endpoint in clinical trials to support the overall efficacy and clinical meaningfulness of the treatment. We partnered with Rhizokids International (https://rhizokids.com), a family advocacy group for RCDP, and Med-Life Discoveries LP, a biopharma company developing a potential therapy for RCDP, to conduct this study. Given the significant neurodevelopmental delay in RCDP, and the crucial role that parents and family caregivers for RCDP play in observing their child’s day-to-day experience, the research team: (1) conducted a literature search on the clinical symptoms of RCDP followed by preliminary interviews with key stakeholders in RCDP; (2) conducted focus groups with family caregivers of children with RCDP to determine meaningful concepts related to construct of QOL in RCDP to develop a pilot survey; (3) used pilot survey data and stakeholder feedback to refine the survey instrument; (4) conducted cognitive interviewing to further refine the survey instrument; and (5) conducted a brief longitudinal study to determine the reliability and internal consistency of the RCDP QOL survey instrument. This research study serves as an important first step in establishing the use of the survey instrument as a reliable clinical outcome assessment for RCDP and more broadly provides a template to standardize the process of developing a QOL survey that appropriately captures the clinical manifestations of rare diseases.

## Methods

### Overall study design and recruitment

**Fig. 1:**
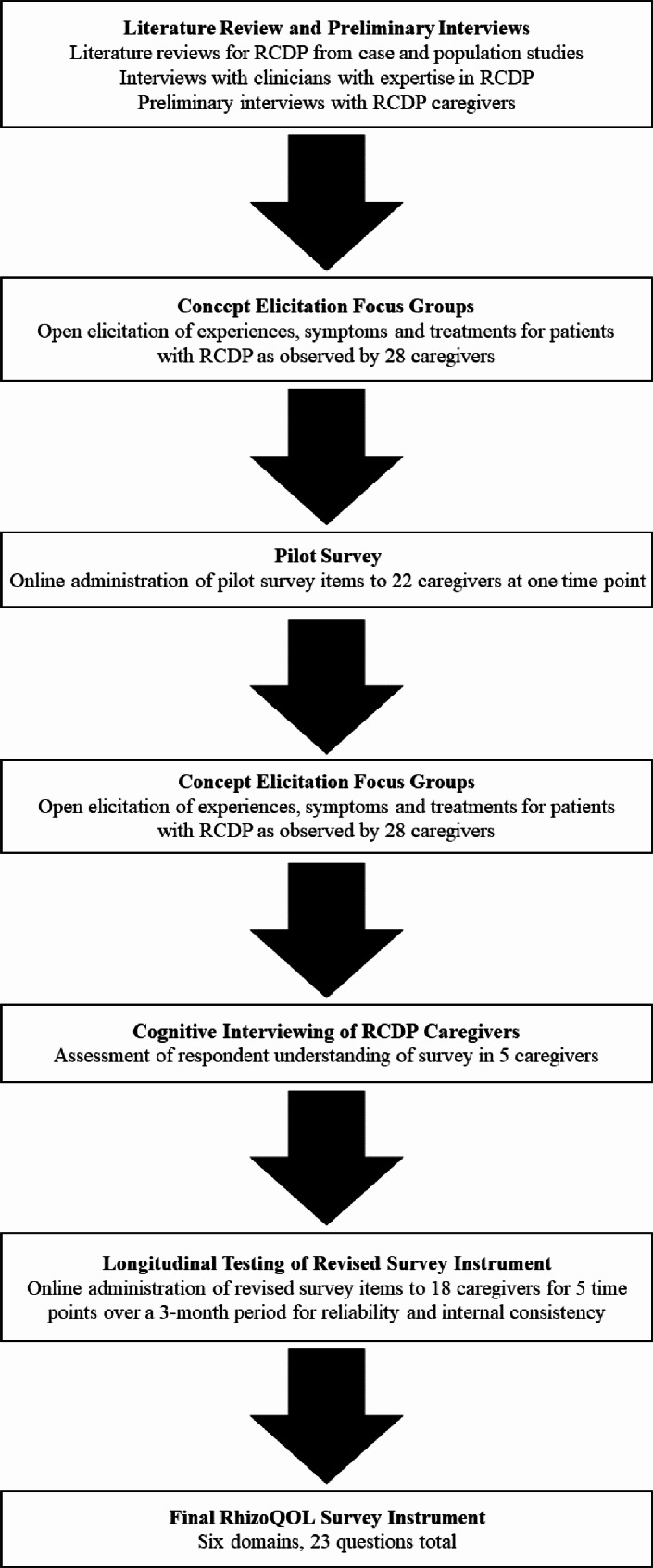
Overview of study chronology for the development of the RhizoQOL survey When it was determined that existing validated survey tools were not appropriate to capture QOL in patients with RCDP, the described tasks were executed in the given timeline. Abbreviations: QOL, quality of life; RCDP, rhizomelic chondrodysplasia punctata

Prior to beginning this study, an extensive search for existing survey tools that could potentially assess QOL in individuals with RCDP was conducted. In addition to relevant literature searches, we surveyed the Patient-Reported Outcomes Measurement Information System (PROMIS) and commercially developed survey tools. Together with key RCDP stakeholders, we determined that none of the existing validated survey tools were appropriate to capture QOL in patients with RCDP and therefore designed and executed our study. Approval for the study was granted by the Montclair State University Institutional Review Board (IRB-FY-17-18-1045). Focus groups, a preliminary cross-sectional survey, and cognitive interviews were conducted to identify symptoms, treatments, and overall experiences of RCDP from the caregivers’ observations. This information was used to generate a draft of the survey instrument which was evaluated for reliability and internal consistency with a brief longitudinal (3 months) study. Based on the findings of the longitudinal study, the survey tool was finalized (See Fig. 1 for overall study chronology). Recruitment flyers were posted on the website and social media page for both RhizoKids International and Med-Life Discoveries at each step of the study to solicit enrollment. RhizoKids International further supported recruitment through e-mail announcements to its members. Individuals self-selected to participate by providing informed consent during each step of the study.

### Development of the QOL focus group interview guide

First, a targeted literature search and review was conducted to identify current clinical research findings in RCDP as a way to comprehensively understand the impact and burden of RCDP. The research team then conducted interviews with stakeholders and key opinion leaders for feedback on a preliminary QOL construct in children with RCDP. This construct was expected to be influenced by various domains related to individual health, but the ultimate intention was for all of the identified domains collectively to serve as an overall composite of QOL in RCDP. A draft of the focus group interview questions was developed based on the preliminary framework. These focus group questions were tested during a conference call that included three members of the research team and five family caregivers of children (both living and deceased) diagnosed with RCDP. During this call, the purpose, goals, and format of data collection were described, including the tentative topics that would be asked during the focus groups. Family caregivers were encouraged to provide feedback on the questions with respect to applicability to their child’s experience with RCDP and were requested to suggest additional questions that might be applicable. A final semi-structured interview guide for the focus groups was developed based on the feedback from the family caregivers and previously published guidance documents [17].

### Concept elicitation focus groups

Inclusion criteria for the focus groups was defined as being a primary family caregiver (parent, stepparent, legal guardian) of a child (living or deceased) with RCDP. All recruited participants had confirmed a genetic and/or biochemical diagnosis of their child via caregiver report in the RhizoKids International membership registry. To optimize the characterization of the full range of the RCDP QOL construct, participation from a broad range of experiences was encouraged (caregivers of children of all ages and symptom presentation, bereaved caregivers, etc.).

#### Data collection from focus groups

Data was collected using a demographic questionnaire and the semi-structured focus group interview guide. The demographic questionnaire gathered information on age, ethnicity, geographical location, family/household information, and diagnoses. It included primarily multiple-choice questions designed to ensure that individual focus groups could be stratified according to specific experiences, such as the age of their child and whether or not their child had passed away.

Three focus groups (8–10 participants per group) were conducted over the course of two days during the RhizoKids International annual conference in July 2018. Each session lasted 90 min and was conducted in English. Sessions were audio and videotaped. The research team lead served as facilitator/moderator. Another research team member served as assistant moderator and took notes on conversations or behaviors not fully captured during the discussions. Notes taken by the assistant moderator were shared with the facilitator during the session to highlight questions that required follow-up and guided the research team in question revision during the focus groups as needed.

To reduce the risk of personal bias from the facilitator and encourage participant-to-participant interactions, the facilitator’s role was limited to reading the questions, distributing response turns, and encouraging personal experience sharing. Individual themes and topics brought up in response to interview questions during the sessions were written on a dry-erase board in the room to keep track of the progression of the discussion. Follow-up prompts to encourage further discussion were used as needed.

#### Data analysis of focus groups

Focus group audio recordings were transcribed verbatim by a professional transcription service *(Rev.com*,* San Francisco*,* CA).* Transcripts were reviewed and response patterns by caregivers were noted, with particular focus on any misinterpretations of the intended meaning of the participants’ words.

The transcripts were analyzed using a combination of deductive (based on the previously established framework) and inductive content analysis techniques [18]. A codebook was developed integrating common themes, as well as words and phrases that caregivers used to describe their observations and experiences. The final codes were cataloged in a qualitative software program *(NVivo Pro version 11*,* QSR International Pty Ltd)*, and used to assign codes to participants’ comments throughout each transcript. The codes and corresponding comments were quantified by frequency, referring to the number of times a comment was categorized into a specific code, and extensiveness, referring to the number of participants that made comments that were categorized into a specific code [19]. For content validity, multiple techniques including peer debriefing and method triangulation were used to ensure that the major themes and sub-themes were generated rigorously and systematically [20–23]. Briefly, three team members met weekly over a period of two months to discuss their experiences with coding. These discussions involved the addition, removal, and merging or splitting of codes based on consensus agreement. Following the completion of coding, team members compared and discussed coding patterns and addressed coding discrepancies, re-evaluating audio recordings and transcript text when necessary. These approaches served to reduce biases and comprehensively generate appropriate concepts to inform a QOL construct for RCDP as observed by the family caregivers.

When a consensus on the pertinent QOL concepts was reached among team members, the team leader re-evaluated the transcripts to assess for concept saturation [15]. This process ruled out the need for additional data collection as well as allowed for prioritization of concepts for inclusion into the RCDP QOL construct.

### Pilot survey development and modification

Draft survey question items were developed to reflect QOL in RCDP as captured by the family caregivers in the focus groups. The pilot survey instrument was incorporated into an online survey platform *(Qualtrics.com*,* Provo*,* UT)* for participant testing from July 2019 to September 2019. Once survey data was collected, survey results, additional respondent feedback, and input from stakeholders in the community, medical field, industry, and regulators were used to modify the survey instrument, including addition, omission, and changes in items and item responses.

### Cognitive interviewing for respondent understanding and interpretation

Following the revision of the pilot survey, the research team conducted five cognitive interviews over video conference with family caregivers of children diagnosed with RCDP to determine respondent understanding and interpretation of the revised pilot survey instrument. Interviews were held in 1.5–2 h sessions to evaluate caregivers’ comprehension of the survey content, length of time to complete the survey, and recall time to answer question items. The cognitive interview also served as an opportunity for caregivers to provide feedback regarding the relevance of survey themes, as well as the formatting and structure of the survey. Briefly, members of the research team gave participants an online link to the revised survey and asked participants to state survey instructions, items, and item responses. After participants reiterated each component of the survey instrument, the research team followed with a series of questions to ensure participant understanding of each component and its intention. Next, the participants were asked to complete the entire survey during the video conference, verbalizing their thoughts as they completed the survey. All interviews were recorded.

### Data analysis of cognitive interviews

Data collected from the cognitive interviews were analyzed for participant understanding and relevance, as described in previously published guidance [15]. Briefly, participant quotes from the verbal probing for each survey item or concept portion of the interview and from the “think aloud” survey portion of the interview were organized in tabular form. Problems with comprehension were evaluated for indications that changes might be needed, and any missing content (including instructions, items, or item responses) identified during the interview was included as potential additions to the instrument. Stakeholders reviewed the analysis table findings and corresponding revisions were made to the survey instrument.

### Longitudinal testing of revised survey instrument for reliability and internal consistency

#### Survey administration and data collection

Once the revised survey instrument was developed, participants were recruited for the longitudinal survey data collection portion of the study. Over three months of data collection, participants were requested to initially complete the survey on three consecutive days then once a month for two months, for a total of five time points per participant. Participants were sent an email at each time point with a link to complete the survey and were instructed to complete the survey within 12 h of the email receipt. Participants were sent a follow-up reminder email if the surveys were not completed within the indicated time frame. Data collection took place from October 2020 to April 2021.

#### Data analysis and subsequent survey modification

Data analysis was performed using IBM SPSS Statistics-version 25 (IBM Corp. Released 2012. IBM SPSS Statistics for Windows, Version 25.0. Armonk, NY: IBM Corp.) Individual survey item responses for each question item were assigned an ordinal value from 1 to 5, with a score of “1” generally indicating a milder impact of RCDP symptoms, and “5” generally indicating a more severe impact of RCDP symptoms. Descriptive data, including mean, standard deviation, median, and minimum and maximum score were tabulated. Cronbach’s alpha was performed to determine the inter-question intra-survey question reliability on a scale of 0 (low reliability) to 1 (high reliability). Cronbach’s alpha scores above 0.65 were considered acceptable [24]. An additional test was performed to determine what Cronbach’s alpha would be following the singular removal of each survey question to determine the relative compatibility of each question’s response output with the collective data set. Linear regression modeling was used to identify which combinations of survey question items most successfully predicted the summed results of the entire survey instrument (per respondent) and the magnitude of this predictive power (R^2^).

The magnitudes of treatment effects required to demonstrate significant improvement following a novel intervention were also determined for each question. To assess this, a modified t-test formula was used to calculate the maximum mean values in a hypothetical post-treatment data set that yielded significant improvement compared to the current dataset. These tests were performed as one-tailed tests, as the focus of the hypothetical post-treatment results was to evaluate the ability to detect a significant reduction in severity (going from a higher to a lower score). In addition to assessing the magnitude of the treatment effect of all participant data, an additional analysis was performed to determine the maximum mean values in a hypothetical post-treatment data set that yielded significant results on a subset of the data, where current survey scores below 2.0 were omitted. The rationale for this subset was that assessment for possible treatment effects in respondents that show only a mild impact of RCDP symptoms (i.e., values below 2.0) in the pre-treatment survey could be considered unwarranted, or even counterproductive noise to the resolving power of the analyses. For all analyses of longitudinal survey data, significance was assigned at *p* < 0.05.

## Results

### Development of the QOL focus group interview guide

The literature review, preliminary interviews, and focus groups contributed to the development of an initial QOL framework composed of concepts identified as contributing factors to QOL in patients diagnosed with RCDP and their families. Eight overall concepts were identified including feeding and diet issues, seizures, orthopedic function, respiratory function, growth and development, psychological function, social function, and family impact. These concepts were used as the foundation to inform the development of the focus group interview guide (Table 1).

**Table 1:** Final semi-structured interview guide for RCDP QOL focus groups

**General** • Tell me about when you first received your child’s diagnosis. • Give me one statement about how RCDP has affected your child’s life. • Give me one statement about how RCDP has affected your life. • Describe a typical day living with RCDP. • Describe a good day living with RCDP. • Describe a bad day living with RCDP.
**If the following topics are prompted by participants during the general question portion, questions from those topics will be asked.**
**Feeding** • Tell me about your child’s feeding experience. What would you consider a good feeding experience? • What are your concerns about your feeding experience? a. If prompted: Fussiness/Discomfort due to feeding i. What does that look like? ii. How do you know when your child is less fussy or more fussy? b. If prompted: Length of time to feed i. What are the factors that increase or decrease the length of time to feed? c. If prompted: GI function with regard to feeding i. What are the GI symptoms that your child experiences due to feeding? 1. Prompt: vomiting, gas, diarrhea, constipation
**Seizures** • Tell me about your child’s experience with seizures. • What are your main concerns about your child having seizures? • How do seizures affect your child’s ability to function? a. If prompted: Do seizures cause pain? What does pain due to seizures look like? b. If prompted: Do seizures affect breathing? Can you tell me how breathing is affected by seizures?
**Orthopedic** • Tell me about your child’s experience with skeletal/muscle issues. • What are your main concerns with orthopedic issues in your child? • How do skeletal/muscle issues affect your child’s ability to function? a. If prompted: Do skeletal/muscle issues cause pain? What does pain due to skeletal/muscle issues look like? b. If prompted: Do skeletal/muscle issues affect mobility?
**Respiratory** • What are your biggest concerns regarding respiratory issues in your child? • How do respiratory issues affect your child’s life? a. If prompted: How do hospital visits related to respiratory infection affect your child’s life?
**Other Physical Aspects** • Tell me about your child’s experience with reaching milestones. How has that impacted their life? • What are the treatments that your child takes due to this disorder? a. How often are treatments needed? b. How much time do you spend during the day administering treatments? c. How do the different medications/treatments affect your child?
**Social** • How does your child show that they are interacting with someone?
**Psychological** • What is your child’s overall mood? What can change your child’s mood? • How do you know your child is happy? What signs do they show? • How do you know your child is upset? What signs do they show? • Are there any other emotions/moods your child experiences that you would like to talk about?
**Family Impact** • Tell me about the impact that this disorder has had on your family life. • If prompted: How has this disorder affected your financial situation? • If prompted: If your child has siblings, how has this disorder affected their lives? • If prompted: How has this affected your relationship with your partner/co-parent? • How would you describe your emotional experience living with RCDP in one word?

### Concept elicitation focus groups

#### Sample demographics and characteristics

The Rhizokids International annual conference included 38 primary caregivers of which a total of 28 subjects (18 mothers and 10 fathers) representing 20 children with RCDP participated in the focus groups. Within this group, 23 participants were caregivers of living children and 5 were caregivers of deceased children. Three focus groups were conducted and arranged by age of proband (either current age or age at the time of death for bereaved caregivers). Based on the attendance and corresponding ages of probands at the conference, Group 1 consisted of caregivers for children with RCDP aged 1–4 years, Group 2 consisted of caregivers for children with RCDP aged 5–7 years, and Group 3 consisted of caregivers for children with RCDP aged 11–13 years. No caregivers of children ages 8–10 years enrolled to participate in the study. Four bereaved caregivers were included in Group 2. One bereaved caregiver for an infant diagnosed with RCDP was included in Group 3 due to a late consent occurring after the previous focus groups had taken place. Most participants in Group 3 were considered “long-term survivors” of the broader RCDP population and presented with generally milder symptoms compared to Groups 1 and 2. Among each focus group, 24 caregivers had one affected child, and 4 caregivers had two affected children (Supplementary Table 1).

#### Concept elicitation, validation, and saturation

**Fig. 2:**
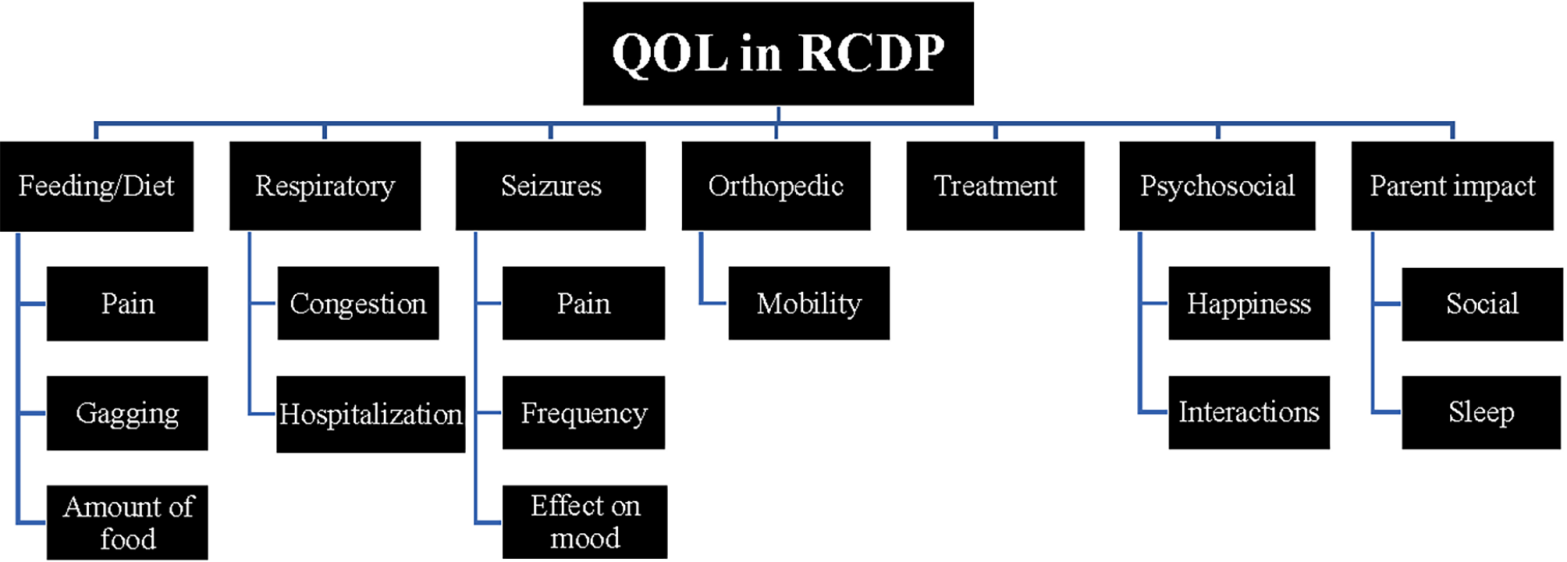
Conceptual framework for QOL in RCDP based on focus groups Abbreviations; QOL, quality of life; RCDP, rhizomelic chondrodysplasia punctata

Analysis of data generated seven themes and concepts related to QOL in children with RCDP. The broad concepts that were initially identified during the focus groups were related to (1) feeding, diet, and gastrointestinal issues; (2) respiratory symptoms; (3) seizures; (4) issues related to orthopedic symptoms; (5) treatment; (6) psychosocial symptoms; and (7) impact on parents. The conceptual framework for QOL was developed to reflect the themes generated by the focus group discussions (Fig. 2).

The presence of emergent concepts associated with QOL, identified by focus participants, is illustrated in the saturation table in which the concepts are compared across all three focus groups (Table 2). We categorized the table by the seven emergent concepts, with each of the 48 sub-concepts thereafter marked if elicited from a particular focus group. Thirty-three sub-concepts were marked as being present in all three focus groups, indicating the majority of symptoms are present across RCDP patients of varying ages. The three focus groups elicited all sub-concepts identified in the psychological and social function and the impact on caregiver domains. There were a small number of new sub-concepts that emerged in the final focus group, including issues with diet composition and nutrition, gastroparesis, respiratory function after experiencing seizures, and pulmonary hypertension. Given that these additional concepts were primarily associated with long-term survivors and no other RCDP subpopulation (confirmed by participant and other stakeholder feedback), no additional focus groups were conducted.

**Table 2:** Concept saturation table for QOL in RCDP focus group sessions

Concept	Focus Group 1	Focus Group 2	Focus Group 3
*Feeding/Diet/ GI Function*			
Pain and Discomfort	X	X	X
Gagging, Vomiting, Reflux	X	X	X
Bowel Movements, Gas	X	X	X
Air Trapped in Stomach	X	X	X
Gastroparesis			X
Frequency Duration of Feeding	X	X	X
Amount of Food Consumed	X	X	X
Weight Concerns	X	X	X
Diet Composition, Nutrition			X
Secondary GI disease		X	
Hospitalization due to GI Issues		X	X
Treatment	X	X	X
*Respiratory Function*			
Infection	X	X	X
Aspiration	X	X	X
Apnea	X	X	X
Airway Collapse	X		X
Pulmonary Hypertension			X
Congestion and Secretions	X	X	X
Hospitalization due to respiratory issues	X	X	X
Treatment	X	X	X
*Orthopedic Function*			
Pain and Discomfort	X	X	X
Kyphosis	X	X	
Mobility	X	X	X
Muscle Tone		X	X
Impact on Respiratory Function		X	
Sleep		X	
Sensory Stimulation	X	X	X
Treatment	X	X	X
*Seizures*			
Pain and Discomfort	X	X	X
Frequency, Duration	X	X	X
Shift in Mood	X	X	X
Sleep, Fatigue	X	X	X
Impact on Respiratory Function			X
Impact on Therapy	X	X	
Triggers	X	X	X
Impact on Milestones		X	
Hospitalization due to seizures	X	X	
Treatment	X	X	X
*General Impact of Treatment*			
Frequency/Duration of Medical Appointments	X	X	X
Use of Medical Equipment	X	X	
Frequency of Medication	X	X	X
Side Effects of Medication and Treatment	X	X	X
*Psychological and Social Function*			
Indicators of Happiness	X	X	X
Communication/Interaction	X	X	X
Independence	X	X	X
*Impact on Caregiver*			
Burden of tasks	X	X	X
Social Interactions	X	X	X
Sleep	X	X	X

Three focus groups (8–10 participants per group) were conducted over the course of two days during the RhizoKids International annual conference in July 2018. Each session lasted 90 min and was conducted in English. Sessions were audio and videotaped. There were 7 emergent concepts during the focus groups, with each of the 48 sub-concepts thereafter marked if elicited from a particular focus group. Thirty-three sub-concepts were marked as being present in all three focus groups. There were a small number of new sub-concepts that emerged in the final focus group. Given that these additional concepts were primarily associated with long-term survivors and no other RCDP subpopulation (confirmed by participant and other stakeholder feedback), no additional focus groups were conducted. Abbreviations; QOL, quality of life; RCDP, Rhizomelic chondrodysplasia punctata; GI, gastrointestinal

Direct quotations from participants describing their observations with respect to QOL in their children diagnosed with RCDP were used to validate emerging concepts and sub-concepts (Table 3). Based on the frequency and extensiveness of quotes related to concepts, issues regarding feeding/dietary intake were the most highly prioritized concepts concerning QOL in RCDP, followed by respiratory function, orthopedic function, and seizures. The feeding/diet/GI function domain referenced issues associated with nutrition and feeding, particularly regarding pain and discomfort during feeding, the frequency and amount of food consumed during feeding, as well as gastrointestinal issues (e.g., bowel movements, gas, constipation). The respiratory function domain included concepts related to blocked airways and concern with breathing (e.g., aspiration, apnea, infection). The orthopedic function domain highlighted symptoms related to pain, discomfort, and muscle tone problems, as well as mobility and its impact on respiratory function and sleep. The seizure domain also described experiences with pain and discomfort and included concepts about the frequency and duration of seizures, triggers, shifts in mood, and treatment. Responses from the general impact of treatment domain referenced frequency, duration, and side effects of medication, the psychological and social function domain referenced indicators of happiness and forms of communication, and the impact on caregiver domain referenced the burden of tasks and the lack of sleep and social interactions. This data was used to inform the development of a pilot survey that included the following domains: (1) psychosocial behavior; (2) feeding symptoms; (3) seizures and myoclonic jerks; (4) other symptoms; (5) hospitalizations; (6) treatment and therapy; and (7) caregiver impact. Each question within the domains had two sets of item responses, one asking for the relative frequency of the event referred to in the question over a period of one week, and the other asking about whether or not that reported frequency was typical of any given week, more frequent than any given week, or less frequent than any given week. Additionally, each domain included one open-ended question, asking the respondent to comment on the relatability of each question item, as well as the readability of the instructions, question items, and item responses (Supplementary Table 2).

**Table 3:** Supporting text of emergent concepts in focus groups

Concept	Focus Group 1 (RCDP patient 1–4 years old)	Focus Group 2 (RCDP patient 5–7 years old)	Focus Group 3 (RCDP patient 11–13 years old)
*Feeding/Diet/ GI Function*			
Pain and Discomfort	“ ***Scream***,*** tense***” “ ***Grunts*** a lot” “ ***Squirming*** around a little bit” “.will try to ***kick his legs***. ***Arching his back***, definitely.” “ Kicking the legs” “ You can usually ***see his veins coming out*** too” “ ***Holding the breath***?” “ Usually after that they’re gone after that, like ***worn themselves out*** to the point”	“it’s always the belly and we went from- it would just be ***screaming***,” “ she’ll start screaming” “It’s, uh, a lot of ***jerking and squirming*** just- just- he just can’t be still” “…you would be halfway through a feed, she would start ***squiggling***,*** grunting***, crying and stuff and I would stop the feed and she would instantly stop crying.”	“Or ***cry***.” “ she’s uncomfortable and crying” “ would ***stiffen up*** and it was so hard to hold her” “ Getting even more stiff” “ ***huge eyebrow movement***” “ with the ***mouth***,*** and then clenching***.”
Gagging, Vomiting, Reflux	“…and ***he might spit up once a day***,*** or once every two to three days instead of two to three times every day***.” “ So a ***good day*** for him would be… ***tolerating his feeds***,*** no spit ups***” “ No spit up” “No reflux” “but ***he would throw up after every feed***,*** and when we fed him in the G… as soon as we had a J we’ve never had a single problem with GI***,*** at all***,*** once***,*** in four years***”	“When it all goes in and stays in.: *Retching* “…doesn’t vomit but he’ll gag. Nothing crazy but ***retching***.”	“…depending on how much she’s tolerating food. Is she gonna throw it up?” “ No puking.” “ ***no throwing up***.” *Retching* “…a ***good day is no gagging***,*** no retching***,”
Bowel Movements, Gas	*Constipation* “…***was constipated for… almost two days***,” “ Probably ***the bad days*** when ***she’s constipated and a little gassy***.”	*Constipation* “And like at least for me like ***we up and down battle constipation***.” *Diarrhea* “…it would just immediately go through her so she- we had to change her and stuff, ***total blowouts***.”	“…and all the ***BM logs*** because they can’t tell you when they’re constipated”
Air Trapped in Stomach	“ she’s swallowing air she’s gets a lot of air in her stomach”	“…and you would feel her stomach and her stomach was real tight, or rock hard”	“It could be physical, like you… ***like a hard belly***”
Frequency Duration of Feeding	“We have to pause, then do it again. ‘Cause she’s bolus, like we were saying. So, ***we usually have to just take more time with it and just go slow.***”	“If I do it faster than 15 min, um, she’ll start screaming and spitting it back up-“	“Or ***shut off the pump during like four times and have to keep resetting***” “…you’re ***constantly having to stop feeding them***”
Amount of Food Consumed	“ They don’t feel good, and then they don’t get a lot to eat so you’re saying like, what’s a clue for me ***that it’s a bad feeding day***,*** I look at the feedbag at the end of the day***,*** the more food that’s in the bag***,*** the worse day she had.*** The less food that’s in there, I know It was a good day.” “…***when they’re comfortable at the time***,*** while they’re eating. I think they’d be more likely to take that and digest it*** and everything”	“So a ***good day was when all the feeds went down well***,*** he took the whole bottle***” “…and ***you gave her too much volume that’s when she had the most discomfort*** and stuff”	“…so ***if I can get the desired amount of food and not have to scale it back***”

Three focus groups (8–10 participants per group) were conducted over the course of two days during the RhizoKids International annual conference in July 2018. Each session lasted 90 min and was conducted in English. Sessions were audio and videotaped. Sample quotes reflecting the most common emotional response based on frequency (number of comments) and extensiveness (number of participants making comments). Abbreviations: RCDP, rhizomelic chondrodysplasia punctata

Data from the pilot survey was collected from 22 caregivers of patients with RCDP at one time point. Using respondent and other stakeholder feedback, a total of 6 domains were used in the revised pilot survey instrument: (1) psychosocial behavior; (2) mobility and orthopedic symptoms; (3) feeding and gastrointestinal symptoms; (4) seizures and myoclonic jerks; (5) respiratory symptoms; and (6) treatment and therapy. For most question items, the responses included an ordinal 5-point Likert scale ranging from “Not at all” [1] to “Always” [5] indicating the occurrence of the symptom/event described in the question item within a period of the last seven days. Within each domain, there was also a survey item that asked caregivers to assess their global impression of the severity of the symptoms in that domain. This was included at the recommendation of regulatory stakeholders. Briefly, participants were asked to rate their observation of the overall severity of that symptom category or rate their level of concern for that symptom category. Item responses included an ordinal 5-point Likert scale ranging from “No symptoms” [1] to “Very Severe” [5] for items assessing severity and from “Not At All Concerned” [1] to “Extremely Concerned” [5] for items assessing level of concern.

### Cognitive interviewing for the revised pilot survey instrument

The coded interview transcripts were analyzed by domain to assess patient understanding of instructions, their responses to each question item, relevancy of questions to their child’s experience with RCDP, suggestions for change, and overall, to see if participants endorsed the content validity of the survey. Participants suggested changes to the wording of question items to make them more relevant to children’s experience with RCDP. Several caregivers mentioned that some questions needed additional language and/or examples to be understood (e.g., including “leftover bolus” in a feeding question or “retched” in a gagging, vomiting, and reflux question).

When conducting the cognitive interviews participants mainly expressed confusion regarding the global index scale questions which assessed their child’s overall experience within a domain. These five questions throughout the survey asked participants to rate their level of concern or the level of severity regarding their child’s experience within a particular domain over the past seven days. Caregivers were asked to explain what aspects of these questions were difficult to understand and they noted that the questions lacked clarity or specificity and made it difficult to interpret what was being asked about their child. One participant explained that the question pertaining to their level of concern regarding their child’s psychosocial behavior over the past seven days was unclear because they assumed that the question was asking about their child’s overall psychosocial behavior, but it was not obvious that this was what the question meant (Supplementary Table 2).

Considering the heterogeneity of the disease, participants noted that even if question items of particular symptoms of RCDP were not relevant to their own child, these questions may still be relevant to other children with RCDP.

*“That they are freely moving their limbs*,* um*,* like for my child he’ll move if he*,* like*,* gets excited. He moves his arms and his legs*,* um*,* you know*,* he doesn’t really use his arm*,* he doesn’t really move his arms and legs a lot*,* so there are children that really do. So*,* him not moving his arms and legs independently is not so much a sign of anything for me*,* but it will be for other families.”*

*“She’s more interactive than I’ve noticed with most children with RCDP. She doesn’t nap like most children do. She likes to lay awake. During the day*,* I see a lot of the children are more tired-looking or just not awake.”*

Suggested changes were reviewed further by the research team using an itemized table, categorized by QOL concepts for each question item participants offered recommendations for. These recommendations were shared with research partners/stakeholders and the survey questions were finalized. Despite some concerns from caregivers regarding clarity on the question items that assessed global impression of severity or level of concern, these questions were included based on regulatory stakeholder feedback. However, instructions to complete these questions were revised to provide more clarity to respondents based on caregiver feedback. A revised survey instrument was developed incorporating all pertinent data from the focus groups as well as feedback from cognitive interviews and stakeholders on draft question items and item responses.

### Longitudinal testing of revised survey instrument for reliability and internal consistency

Longitudinal survey data was comprised of 18 participants responding to 31 question items on each of five separate days. For each of the question items, a total of 74 data points were collected. The data was analyzed for descriptive statistics, intra-survey question reliability, linear regression modeling, and required magnitude of treatment effects. All item responses were ordinal responses on a scale of 1 to 5. For 27 of the 31 question item responses, a higher numeric value was indicative of a more unfavorable impact of the symptoms/events in the question item. In order to streamline the data analysis, the item responses for the remaining four questions were inverted to accomplish a uniform scale of 1 = lowest severity/most favorable and 5 = greatest severity/least favorable for all question items.

The average mean score across all question items was 1.98 ± 1.0, showing overall that survey scores are closer to the floor value of 1 than to the ceiling value of 5. When stratified by domain, the domain assessing the impact of feeding/gastrointestinal symptoms showed the highest average mean score (2.16 ± 1.1), while the domain assessing the impact of treatment and medical interventions showed the lowest average mean score (1.47 ± 0.7). For question items assessing the global impression of severity or concern, the average mean score was 1.85 ± 0.3, while the average mean score of all question items EXCEPT those assessing for global impression of severity or concern was 2.02 ± 1.0 (Table 4).

**Table 4:** Mean, minimum, and maximum scores of longitudinal survey by domain (*N* = 74 responses total)

DOMAIN	Mean	Minimum	Maximum
Psychosocial	1.64 ± 0.6	1.00	3.33
Mobility and Orthopedic	2.15±0.8	1.00	3.75
Feeding/GI	2.16±1.1	1.00	4.71
Seizures	2.09±1.1	1.00	4.57
Respiratory	2.06±1.2	1.00	4.83
Treatments/Interventions	1.47±0.7	1.00	3.50
All CGI Items	1.85±0.7	1.00	3.83
All Domains WITHOUT CGI Items	2.02±1.0	1.00	4.40
**All Items**	**1.98±1.0**	**1.00**	**4.83**

Longitudinal survey data consisted of 18 participants responding to 31 question items on each of five separate days. For each of the question items, a total of 74 data points were collected. Abbreviations: CGI, Caregiver Global Impression of Severity or Concern

The Cronbach’s alpha test for intra-survey question reliability for the survey instrument was 0.856, which was above the predetermined acceptability cut-off of 0.65, suggesting good intra-survey question reliability. Cronbach’s alpha values were also generated following the removal of each of the survey question items to determine the relative compatibility of each question item response output with the collective score from all question items (Table 5). It was determined that individual removal of four specific question items within the domain assessing the impact of seizures in RCDP resulted in an increase of Cronbach’s alpha values for the survey instrument. These question items were related to the frequency of seizures, and how often a patient indicated discomfort/pain, participated in daily activities, or showed a worsening in mood following a seizure. The removal of two specific question items within the domain assessing the impact of feeding symptoms in RCDP resulted in an increase of Cronbach’s alpha values for the survey instrument. These question items were related to how often a patient did not complete a meal or had difficulties related to constipation. The removal of one question item regarding the need for respiratory support in the domain assessing the impact of respiratory symptoms in RCDP resulted in an increase of Cronbach’s alpha values for the survey instrument. There were no individual question items that assessed the global impression of severity or concern in the domains that resulted in a higher Cronbach’s alpha score upon its removal from the survey instrument.

Linear regression modeling analysis was used to determine which combinations of survey question items most successfully predicted the results from the scores of the entire survey instrument. A single question item related to the overall global impression of respiratory symptom severity predicted 59.1% of the variance observed for the completion of the entire survey instrument. Inclusion of four other question items, two assessing the global impression of severity of mobility symptoms and seizures, respectively, one assessing the frequency of air retention in the gastrointestinal tract during feeding, and one assessing the frequency of contacting medical providers predicted 92.2% of the variance observed for the completion of the entire survey instrument.

A hypothetical post-treatment data set was generated to determine whether the question items could be used to detect a significant improvement with treatment. The hypothetical mean post-treatment response for the entire survey instrument would be 1.45 or below, and the average treatment effect required would be 0.54. A total of 29 of the 31 question items could theoretically yield a significant post-treatment effect. The remaining two question items (one about the frequency of seizures being triggered by fever and one question assessing the frequency of treatment/medication used to treat acute respiratory symptoms) required a hypothetical post-treatment score of less than 1 to demonstrate a significant treatment effect, which would not be possible (Table 5).

**Table 5:** Statistical analyses of longitudinal data collection for RCDP QOL survey

Question Item	Mean Score ± SD	Score Required for Significant Treatment Effect	Cronbach’s 𝛂 value if question item was removed	Omit/Keep Item
**All Question Items**	**1.98±1.0**	**1.45**	0.856*	**N/A**
Psychosocial Domain - Item 1	1.85 ± 0.8	1.42	0.850	Keep
Psychosocial Domain - Item 2	1.88 ± 0.5	1.63	0.851	Keep
Psychosocial Domain - CGI	1.18 ± 0.4	1.02	0.853	Keep
Mobility Domain - Item 1	2.56 ± 0.9	2.00	0.850	Keep
Mobility Domain - Item 2	2.24 ± 0.8	1.82	0.853	Keep
Mobility Domain - Item 3	1.79 ± 0.7	1.39	0.849	Keep
Mobility Domain - CGI	2.02 ± 0.7	1.68	0.851	Keep
Feeding/GI Domain - Item 1	2.12 ± 1.2	1.49	0.858	Keep
Feeding/ GI Domain - Item 2	1.91 ± 1.0	1.32	0.849	Keep
Feeding/GI Domain - Item 3	2.30 ± 1.1	1.67	0.848	Keep
Feeding/GI Domain - Item 4	1.95 ± 1.1	1.34	0.859	Keep
Feeding/GI Domain - Item 5	1.84 ± 1.3	1.07	0.861	Omit
Feeding/GI Domain - Item 6	2.83 ± 1.4	2.01	0.846	Keep
Feeding/GI Domain - CGI	2.19 ± 0.8	1.76	0.845	Keep
Seizures Domain - Item 1	3.03 ± 1.2	2.30	0.864	Keep
Seizures Domain - Item 2	2.00 ± 1.2	1.32	0.845	Keep
Seizures Domain - Item 3	1.20 ± 0.6	0.90	0.852	Omit
Seizures Domain - Item 4	2.17 ± 1.2	1.47	0.859	Omit
Seizures Domain - Item 5	2.07 ± 1.4	1.31	0.867	Omit
Seizures Domain - Item 6	1.95 ± 1.1	1.29	0.859	Keep
Seizures Domain - CGI	2.21 ± 0.8	1.75	0.853	Keep
Respiratory Domain - Item 1	3.34 ± 1.1	2.79	0.846	Keep
Respiratory Domain - Item 2	1.29 ± 0.6	1.05	0.850	Omit
Respiratory Domain - Item 3	2.24 ± 1.5	1.30	0.842	Omit
Respiratory Domain - Item 4	2.00 ± 1.6	1.00	0.857	Keep
Respiratory Domain - Item 5	1.47 ± 1.1	0.88	0.849	Omit
Respiratory Domain - CGI	2.00 ± 1.0	1.48	0.842	Keep
Treatments Domain - Item 1	1.63 ± 0.9	1.13	0.848	Keep
Treatments Domain - Item 2	1.46 ± 0,7	1.11	0.847	Omit
Treatments Domain - Item 3	1.33 ± 0.6	1.05	0.850	Keep
Treatments Domain - CGI	1.47 ± 0.7	1.14	0.849	Keep

A hypothetical post-treatment data set was derived from the longitudinal survey data to determine whether the question items could be used to detect a significant improvement in treatment. A total of 29 of the 31 question items could theoretically yield a feasible (greater than a score of 1) and significant post-treatment effect. Abbreviations: CGI, Caregiver Global Impression of Severity or Concern

A second analysis was performed with a subset of data in which question item responses with a score less than 2.0 were omitted. In this case, the pre-treatment mean was 2.94, with the hypothetical mean post-treatment response for the entire survey instrument at 2.35 or below, and the average treatment effect required would be 0.59. Twenty-six of the 31 question items could theoretically detect a significant post-treatment effect. In addition, the two question item responses that failed to show a feasible significant treatment effect in the complete dataset (one global impression question item assessing the caregiver’s level of concern regarding their child’s psychosocial behavior) did not have enough data in this subset to perform the analysis (Supplementary Table 3).

### Final RhizoQOL survey instrument

Based on the results of the longitudinal testing data, eight question items from the draft survey instrument were removed and the final survey instrument called “RhizoQOL” was generated. Among the removed question items, three were related to seizure activity, three were related to respiratory symptoms, one was related to gastrointestinal symptoms, and one was related to treatments and interventions. The final survey instrument includes 23 question items divided into six domains. Three question items are related to psychosocial symptoms, four are related to mobility symptoms, six are related to gastrointestinal symptoms, four are related to seizure activity, three are related to respiratory symptoms, and three are related to treatment and interventions. Each of these domains also includes a global impression question item (Table 6).

**Table 6:** Final domains and sample question items/responses included in RhizoQOL survey instrument

DOMAIN	CGI Question Item	Item Responses
PSYCHOSOCIAL BEHAVIORS	My overall level of concern regarding my child’s psychosocial behavior over the past 7 days has been:	Not at All Concerned, Slightly Concerned, Somewhat Concerned, Moderately Concerned, Extremely Concerned
MOBILITY AND ORTHOPEDIC SYMPTOMS	The overall level of severity regarding my child’s mobility and orthopedic symptoms over the past 7 days has been:	No Symptoms, Not Severe, Mildly Severe, Severe, Very Severe
FEEDING AND GASTROINTESTINAL SYMPTOMS	The overall level of severity regarding my child’s feeding and gastrointestinal symptoms over the past 7 days has been:	No Symptoms, Not Severe, Mildly Severe, Severe, Very Severe
SEIZURES	The overall level of severity regarding my child’s seizures over the past 7 days has been:	No Symptoms, Not Severe, Mildly Severe, Severe, Very Severe
RESPIRATORY SYMPTOMS	The overall level of severity regarding my child’s respiratory symptoms over the past 7 days has been:	No Symptoms, Not Severe, Mildly Severe, Severe, Very Severe
TREATMENT AND MEDICAL INTERVENTIONS	My child has been bothered by treatment or medical interventions:	Not at all, A little bit, Somewhat, Quite a bit, Very much

The final RhizoQOL survey instrument includes 23 question items divided into six domains. Three question items are related to psychosocial symptoms, four related to mobility symptoms, six related to gastrointestinal symptoms, four related to seizure activity, three related to respiratory symptoms, and three are related to treatment and interventions. Each of these domains also includes a caregiver global impression question item. Abbreviations: CGI, Caregiver Global Impression of Severity or Concern

## Discussion

The goal of this project was to find a tool that could assess the QOL of children with RCDP. The team first considered whether it would be possible to repurpose an existing validated survey tool and incorporate it into a therapeutic clinical trial for RCDP as a clinical outcome assessment. Despite an extensive search, it was determined that existing tools could not reliably serve in this capacity, therefore we sought to undertake the steps necessary to develop an original caregiver-reported QOL survey instrument specifically for patients with RCDP. Throughout our focus groups, preliminary survey study, and cognitive interviews, our team identified feeding/gastrointestinal issues, respiratory symptoms, mobility and orthopedic symptoms, seizures, impacts of treatment, and psychosocial symptoms as key concepts that influence QOL in patients diagnosed with RCDP. Respiratory symptoms, mobility, orthopedic symptoms, and seizures have all been previously characterized in RCDP [7, 8, 10], as well as cervical spine deformities (e.g., spinal stenosis) [9].

To our knowledge, this is the first report describing gastrointestinal symptoms as part of the RCDP symptomatology. One study has reported gastrostomy tube feeding in 65% of studied patients (*n* = 12) [6] and an older case study showed feeding difficulties in a patient with RCDP [25], however, these difficulties have been primarily attributed to oral motor dysfunction rather than gastrointestinal symptoms [7]. Gastrointestinal symptoms, psychosocial symptoms, and impacts of treatment are known to be key concepts for QOL in other chronic disorders. The fact that these domains factored strongly in the initial QOL discussions within this population provided confidence in the construct validity.

The pilot testing results and coded interview transcripts from the cognitive interviews were analyzed to assess patient understanding of instructions, their responses to each question item, relevancy of questions to their child’s experience with RCDP, suggestions for change, and overall, to see if participants endorsed the content validity of the survey. Participants suggested changes to the wording of question items to make them more relevant to children’s experience with RCDP. Several caregivers mentioned that some questions needed additional language and/or examples to be understood (e.g., including “leftover bolus” in a feeding question or “retched” in a gagging, vomiting, and reflux question). Throughout this evaluation process, we incorporated changes, additions, and removals in our instructions, question items, and item responses. While the clinical presentation of RCDP varies widely, we believe the final survey instrument is capable of capturing an appropriate composite index of the impact of RCDP on activities of daily life. Further, we believe that incorporating such a tool in an interventional RCDP trial will be instrumental in supporting whether the investigational treatment provides a meaningful improvement in the overall QOL of individuals with RCDP.

We were given stakeholder feedback to include question items within each domain that assessed a caregiver’s global impression of severity or concern. These six global impression question items throughout the survey asked participants to rate their level of concern or the level of severity regarding their child’s experience within a particular domain over the past seven days. Global impression scales are commonly used survey tools in clinical assessments, research, and clinical trials to evaluate the survey respondents’ perception of the severity of a disease, condition, or a specific aspect of the condition. Similarly, global impression of change scales are tools to assess the survey respondents’ perception of improvement or worsening following the implementation of a treatment in a clinical trial or other research [26]. Historically, the respondents to these instruments have been clinicians, based on their assessment of a patient under their care [27]. Over the last decade, there has been one study that developed a global impression scale of severity for primary caregivers of patients diagnosed with schizophrenia [28], and multiple studies have developed and utilized global impression of change scales when assessing the effectiveness of treatments in various rare diseases [29–31]. When conducting the cognitive interviews, participants mainly expressed confusion regarding the global impression of severity or concern question items which assessed their child’s overall experience within a domain. We asked caregivers to explain what aspects of these questions were difficult to understand; they noted that the questions lacked clarity or specificity and made it difficult to interpret what was being asked about their child. In our longitudinal data analyses, however, the inclusion of these global impression question items appeared to improve the reliability of the survey instrument, determined by Cronbach’s alpha analysis. Additionally, each of the global impression question items could potentially yield a feasible and significant treatment effect given the initial longitudinal data. Based on this evidence, and the increasing use of these scales in clinical trials, we included each global impression of severity question item in the final survey instrument. Currently, the RhizoQOL instrument is included in the ongoing, prospective RCDP Natural History Study (ClinicalTrials.gov ID NCT04031287). Future analysis of the longitudinal data gathered on this larger group of RCDP patients will allow us to confirm the reliability of the global impression of severity questions by comparing the responses to related changes in other clinical assessments included in the natural history study. In addition, future studies could include interviews with the natural history study participants caregivers to gain insight into the clarity of the global impression scores and if necessary, refinements to the wording could be implemented.

The majority of recall periods used in disease-specific patient-reported clinical outcomes assessments, such as QOL surveys, range from one day to one month. We chose a recall period of seven days as this recall period has been shown to be justified when assessing for concepts such as gastrointestinal symptoms and orthopedic function, similar to concepts explored in the RhizoQOL [32]. We expect this seven-day recall period to be appropriate for our expected population, minimizing both the risk of missing symptoms and impacts that do not occur on a daily basis, as well that of relying too heavily on memory for a longer recall period.

Our longitudinal study statistical analyses found that the Cronbach’s alpha value for the entire survey instrument was 0.856. A Cronbach’s alpha value of 0.65–0.80 is conventionally considered “adequate” for a scale used in human dimensions research [24]. Of the eight question items that were removed from the survey, the removal of three of those question items showed an increase in the Cronbach’s alpha value. Taken together, these data suggest favorable inter-question intra-survey question reliability in our final survey.

Two question items were removed from the survey instrument due to their failure to yield a feasible and significant treatment effect based on the initial longitudinal data. Additionally, three question items were removed from the survey instrument as the vast majority of respondents scored these question items below 2.0, suggesting it would not be useful in detecting improvements with the introduction of treatment. Overall, we removed questions from the survey instrument to increase the intra-survey reliability and the likelihood of detecting a significant treatment effect, if present in a future clinical trial.

Our RhizoQOL survey instrument consists of 23 questions, with an estimated completion time of 12–13 min. Guidance on survey development in healthcare outcomes research generally recommends an estimated completion time of 15–20 min to reduce burden on the participant. Our survey falls below this recommended time which was important to the team given the high burden RCDP caregivers already face.

The present study does have some limitations. First, demographic information was only collected during the focus group phase of the study. In the focus groups, caregiver demographic characteristics (including race/ethnicity, education, and socioeconomic status) were fairly homogeneous with the exception of gender. Although a broad range of topics were discussed in the focus groups, there may be additional issues of importance to caregivers that were missed given the demographic similarities across the groups. Future recruitment efforts in the RCDP Natural History Study, as well as related studies, will need to be further targeted to ensure inclusion of participants from a variety of backgrounds.

Survey scoring for the RhizoQOL rates a better QOL with a lower overall score, and a worse QOL with a higher overall score. This is in contrast to many health-related QOL survey instruments, which generally attribute a better QOL with a higher score. However, disease-specific scales, such as ours, often rely on the presence of disease-related events, as opposed to the absence of these events. As a result, the score for scales such as these is often based on frequency and severity of these disease-specific events, yielding a higher number that correlates with a lower overall QOL [33, 34]. Given these established precedents, in addition to our inclusion of the global index of severity question item for each domain, our scoring directionality for the RhizoQOL is reasonable for a disease-specific QOL survey instrument.

Although many caregivers provided comprehensive input on appropriate domains for a quality of life survey in RCDP, and how impactful these symptom domains were on their child’s daily life activities, one of the biggest challenges in this study is that our longitudinal data analyses showed an average score response of less than 2. With a scale of 1–5 for all item responses, where 1 represents least severe and 5 represents most severe, the longitudinal data suggests that, for the domains assessed in this survey instrument, the impact of RCDP is not considered severe, which contradicts what was reported by caregivers in the focus groups. While the responses were less severe than anticipated, the results of the linear regression models show that the majority of the variance in the survey was captured by the caregivers impression of respiratory symptoms, followed by seizure, mobility, and gastrointestinal issues during feeding. This finding aligns well with how the caregivers reported aspects of QOL in the focus groups, supporting the validity of the survey contents.

Another challenge to this study is that, given this hypothetical baseline data, there may not be sufficient room to move the item responses down in response to treatment unless the impact of treatment was profound. While this limits the utility of this survey instrument as a standalone endpoint, it does not negate the importance of its use as an exploratory endpoint. Domain-specific QOL scores could also be compared to domain-specific clinical endpoints to support observed improvements. In addition, while a statistically significant change may not be feasible, an overall trend of improvement on the RhizoQOL would provide strong support for the clinical meaningfulness of changes observed in primary or secondary endpoints.

Studies with family caregivers of rare diseases have indicated that coping strategies, both problem-focused and emotion-focused, are important tools in helping individuals manage their and their child’s daily life activities [35, 36]. Both of these coping strategies have been associated with a higher QOL in human diseases, including rare diseases, and may explain the relatively low score (better QOL) as reported by caregivers in our study [37–39]. Future studies utilizing our survey tool may need to control for coping strategies and factors that predict coping strategies in survey respondents.

Depending on the phase of the study, our sample size ranged from 5 to 28 participants, with broad homogeneity with respect to participant sample demographics. The small sample size is expected for an ultra-rare disease such as RCDP, and this limitation affected our ability to organize focus groups with similar composition concerning demographics and disease presentation. This likely contributed to the emergence of new concepts in our final focus group. Nevertheless, we feel that the concepts included in the QOL construct that we have developed will be reflective of the experience of the majority of RCDP patients. Additionally, although RCDP hypothetically affects all races and ethnic groups, most reported patients are from the United States and Europe. However, future studies should consider how to increase recruitment to include a more demographically diverse population.

This newly developed RCDP QOL survey instrument is a disease-specific scale for assessing the impact of RCDP symptoms, experiences, and care on the daily life activities of patients as observed by their family caregivers. Qualitative feedback from caregivers has indicated satisfactory content validity, while longitudinal data collection has provided psychometric validation on the robustness, reliability, and utility of the instrument, despite some limitations. Although continued research and data collection is warranted, this survey instrument represents a promising tool for clinical trials to assess the overall effectiveness of therapeutics for RCDP.

## Electronic supplementary material

Below is the link to the electronic supplementary material.

Supplementary Material 1: Supplementary Table 1: Demographic data for focus group participants. Demographic information on age, ethnicity, geographic location, family/household information, education, and diagnoses for focus group participants

Supplementary Material 2: Supplementary Table 2: Supporting text of cognitive interviews. Subject responses to question items, subject responses to item response choices, and suggestions for changes to question items.

Supplementary Material 3: Supplementary Table 3: Required magnitude of treatment effect when response scores less than 2.0 are omitted. Hypothetical post-treatment scores based on longitudinal data response scores that are 2.0 or above

## Data Availability

The datasets used and/or analyzed during the current study are available from the corresponding author on reasonable request. Access to the full RhizoQOL survey instrument, as well as further information on, or permission to use, the RhizoQOL can be obtained by contacting Dr. Tara Smith at t.smith@med-life.ca.

## Abbreviations

PROMIS: Patient-reported outcomes measurement information system
QOL: Quality of life
RCDP: Rhizomelic chondrodysplasia punctata
VLCFA: Very long-chain fatty acids
