# Supplementary material for: Development and evaluation of RhizoQOL, a quality-of-life caregiver-reported survey for rhizomelic chondrodysplasia punctata, a rare peroxisomal disorder
Source: Orphanet J Rare Dis. 2025 Mar 31;20:147. doi: 10.1186/s13023-025-03660-0 (PMC11956500; doi:10.1186/s13023-025-03660-0)
Supplement: Supplementary file 1 — Supplementary Material 1: Supplementary Table 1: Demographic data for focus group participants. Demographic information on age, ethnicity, geographic location, family/household information, education, and diagnoses for focus group participants [file 13023_2025_3660_MOESM1_ESM.pdf]

Supplementary Table 1: Demographic data for focus group participants

**Total**	**N**<br>**28**	**%**<br>**100**
**Family Role**		
Mother<br>Father<br>Step Parent	18<br>9<br>1	64.3<br>32.1<br>3.6
**Age (years)**		
25-34	16	57.1
35-44	7	25.0
45-54	4	14.3
55-64	1	3.6
**Ethnicity**		
White	27	96.4
Declined to Answer	1	3.6
**Marital Status**		
Single	0	0.0
Married	28	100.0
**Highest Education**		
Technical	1	3.6
High School Grad	7	25.0
Some College	7	25.0
Bachelor's	8	28.6
Grad/Professional Degree	2	7.1
High School Grad and Some College	1	3.6
Some High School, No GED	1	3.6
Graduate	1	3.6
**Employment Status**		
Self-Employed	2	7.1
Unable to work	3	10.7
Part-Time	2	7.1
Full-Time	13	46.4
Retired	1	3.6
Homemaker/Unable to work	6	21.5
Declined to Answer	1	3.6
**Household Income ($)**		
30-49K	3	10.7
50-69K	4	14.3
70-89K	5	17.9

>90K	11	39.3
Decline	5	17.9
**Region of Residence**		
Midwest	8	28.6
Southeast	14	50.0
Southwest	1	3.6
West	4	14.3
Outside US	1	3.6
**Number of Affected Children**		
1	24	85.7
2	4	14.3
**Status of Child(ren) (Alive/ Deceased)**		
Alive	23	82.1
Deceased	5	17.9
**Age Range of Affected Children (years)**		
<1	2	7.1
1-4	14	50.0
5-7	4	14.3
8-10	3	10.7
11-13	3	10.7
14-17	2	7.1
