# Supplementary material for: Development and evaluation of RhizoQOL, a quality-of-life caregiver-reported survey for rhizomelic chondrodysplasia punctata, a rare peroxisomal disorder
Source: Orphanet J Rare Dis. 2025 Mar 31;20:147. doi: 10.1186/s13023-025-03660-0 (PMC11956500; doi:10.1186/s13023-025-03660-0)
Supplement: Supplementary file 2 — Supplementary Material 2: Supplementary Table 2: Supporting text of cognitive interviews. Subject responses to question items, subject responses to item response choices, and suggestions for changes to question items. [file 13023_2025_3660_MOESM2_ESM.pdf]

Supplementary Table 2: Supporting text of cognitive interviews

Domain	Global Index of Severity	Subject responses to inquiry about what instruction/item means/any difficulty with meaning	Subject responses to item response choices	Suggestion for changes to item (action to take)
**Psychosocial Behavior**	My level of concern regarding my child's psychosocial behavior over the past 7 days has been	Participant 1:<br>"I guess, like, if my child's behavior over the last 7 days, like all of the things that are, I guess clues for me to watch, I guess if it's been typical or if it's been off. I guess is how I would take that to mean."<br><br>What psychosocial behavior means to her, in respect to her son's psychosocial behavior: "Interactions. Like, I guess, when he hears somebody, is he looking for them? When he hears us laughing, does he laugh with us? Those are kind of the things, like, how he interacts with us, how he responds to things, noises, stimulus, all that kind of stuff.<br><br>Participant 2:<br>"Just his overall behavior, how he's been acting."<br><br>Participant 3:<br>"Just if I have any concerns over her behavior that she's had in the week."<br><br>Participant 4:	Participant 1:<br>"I would choose 'Not At All Concerned'.<br><br>To choose 'Extremely Concerned': "I would say that he was sick, he was not responsive, he was lethargic, um, he was not interacting, no response. You know like, if we're talking to him, he's not responding. That would be, like, extremely concerned for us."<br><br>Participant 2:<br>"Not at all concerned."<br><br>Participant 3:<br>"Slightly concerned."<br><br>To choose 'Extremely Concerned': "I guess where she would have multiple seizures in a week's time where she just wasn't really doing well afterwards that we were on a trip to the doctor."<br><br>To choose 'Not at all concerned': "She just had a very relaxed week. Not a lot overstimulated from the environment and stuff."<br><br>Participant 4:<br>"I would say not at all concerned over the past seven days. She's had a really good week as far as interacting with us and	**Participant 4**: "That question may not be quite as clear, although I know it's getting at the previous two items. So the suggestion I would have on that is maybe to make some sort of statement regarding ... I assume what you're getting at is given those two indicators, given those two previous items that you were asked about, what is your overall level of concern?"<br><br>**Moderator**: "You think we should include the word "overall" in that question?"<br><br>**Participant 4**: "I would think so"<br><br>**Researcher**: "One thing that I was saying before is that "overall" is in the instructions, the overall concern or [crosstalk 00:19:46]. But I think including that here, within this question, that would be helpful."<br><br>**Moderator**: "Yes, I did. I knew I had "overall" somewhere. So yes, we'll make sure that we add in the actual placement of the question itself, not just in the instructions. Thank you for that feedback."<br><br>**Change to** --> My overall level of concern regarding my child's psychosocial

		"How would I rate my concerns based on her social behaviors, social interactions, over the past seven days."<br><br>Participant 5:<br>"It's asking me to rate my observation of if I'm concerned or not with her behavior in the past seven days."	other people in the house, and she's been smiling and laughing often and has just been doing really well."<br>To choose 'Extremely concerned': "If we were very concerned with psychosocial behaviors, then we're going to be doing things like taking her to the doctor, we're going to be doing things like trying to get on the phone with any of her specialists, depending on what we think is going on with her. Just trying to figure out what's going on because that's a big indicator for us that something's wrong, either health-wise or something's going on."<br><br>Participant 5:<br>"I would say slightly concerned because I know what's going on. I know that she's going to be getting her periods so that's making her cranky. I know that she's having her stomach issues."<br><br>To choose 'Extremely Concerned': "That she would have to go to the hospital. That she was either throwing up and not being able to hold her feeds because she aspirates and she can't take any food by mouth. That would be concerning because then we would have to worry about her dehydrating."<br><br>To choose 'Not at all Concerned': "Not at all concerned would be that she was really extremely happy and not cranky like she has been and not trying to sleep during the day because she's up all night fussy. Me not having to give her Tylenol or something to help her out and not have to change her medications. Her not twitching	behavior over the past 7 days has been

			as much and having these crazy seizures where her eyes are rolling back in her head and she's just going pale from the seizure."	
**Mobility and Orthopedic Symptoms**	The level of severity regarding my child's mobility and orthopedic symptoms over the past 7 days has been	Participant 1:<br>"In a survey like this, you know cause we've already gone through some of it, it would be above what his typical symptoms would be or like what his average issues are. Like is he having anything above what our norm is?"<br><br>Participant 2:<br>"Sometimes he will quit interacting or smiling or whatever from doing it. If we've done it so much, my child quits or sometimes it does throw him into more seizures so we have to quit."<br><br>Participant 3:<br>"Has she had any pain or issues with mobility or bony things."<br><br>"Has she had any pain or issues with her movement over the past seven days."<br><br>Participant 4:<br>"How severe are the problems that we have with my child's mobility or any type of joint issues?"<br><br>Participant 5:	Participant 1:<br>"I'm thinking pain, like pain when I sit him up. There are times that we have issues with his hips and he can't sit up or, um, moving him rearranging him when I'm doing his like therapy. If I notice that there's like a negative reaction to that. That would be the things I would think of."<br><br>"We haven't had any symptoms over the past 7 days."<br><br>To choose 'Very Severe': "It would be he's got a fever, he's aching. Something's hurting him, he's not feeling good, he's getting sick. That would be 'Very Severe'."<br><br>Participant 2:<br>"I think not severe."<br><br>Would choose very severe if he had more seizures."<br><br>"Participant 3:"<br>My answer would probably be no symptoms."<br><br>To choose 'Very severe': "I guess if she was having more seizures I would choose more severe, because it would hinder her mobility or if she was having arthritic pain, I guess you would say, like in our	Considering adding question item, 'if you try to do some sort of purposeful movement and your child tightened up or grimaced or cried during that movement'.<br><br>Consider directionality of this question.

		"So it's asking me what the rate of from no symptoms to severe or majorly severe of what I've observed the last seven days of her mobility, like moving of her arms and her legs, orthopedically, of that."	shoulders or something like that."<br><br>Participant 4:<br>"I would say not severe, just because I don't think just due to the nature of RCDP, I don't feel like no symptoms is ever going to be a choice that I would give."<br><br>To choose 'Very severe': "We would be very concerned and again, would be in touch with her doctors to see what's going on, how can we help her manage whatever she's experiencing. That type of situation."<br><br>Participant 5:<br>"Over the last seven days it would be in between mildly severe and severe because of her arms bleeding."<br><br>To choose 'Very Severe': "I would be taking her probably to the emergency room because I wouldn't be able to treat it myself and it'd be getting worse and not just cracking and bleeding, but really oozing and maybe having an infection, smelling like an infection, something that I couldn't personally treat."<br><br>To choose 'No Symptoms': "Her arms would look normal. We would just be putting on Aquaphor just to add a little slickness, like with it moving and just treating it as normal."	
**Feeding and Gastrointestinal**	The level of severity regarding my child's feeding and	Participant 1:<br>"How concerned have I been over his stomach issues over the last 7 days. If there were	"Participant 1:<br>"How concerned have I been over his stomach issues over the last 7 days. If there were any indicators that something	

	gastrointestinal symptoms over the past 7 days has been	any indicators that something was going on."<br><br>Participant 1:<br>"No symptoms' No concerning symptoms. No symptoms from issues."<br><br>To choose 'Very Severe': "Definitely if he's not feeling good or he's getting sick, his GI tract slows down."<br><br>"Participant 2:<br>"If his eating and gastro symptoms have been severe or not."<br><br>"Participant 2:<br>"I would say not severe."<br><br>To choose 'Always': "I'm constantly having to slow his rate, or he's constantly gagging or retching, or he's constantly getting vented, then I would choose always."<br><br>"Participant 3:<br>"Has she had vomiting, or has she had trouble feeding, or has she been in pain? Just overall, her feeding issues of the past seven days."<br><br>"Participant 3:<br>"Mildly severe."<br><br>To choose 'No symptoms': "No	was going on."<br><br>"Participant 1:<br>"No symptoms' No concerning symptoms. No symptoms from issues."<br><br>To choose 'Very Severe': "Definitely if he's not feeling good or he's getting sick, his GI tract slows down."<br><br>"Participant 2:<br>"If his eating and gastro symptoms have been severe or not."<br><br>"Participant 2:<br>"I would say not severe."<br><br>To choose 'Always': "I'm constantly having to slow his rate, or he's constantly gagging or retching, or he's constantly getting vented, then I would choose always."<br><br>"Participant 3:<br>"Has she had vomiting, or has she had trouble feeding, or has she been in pain? Just overall, her feeding issues of the past seven days."<br><br>"Participant 3:<br>"Mildly severe."<br><br>To choose 'No symptoms': "No reflux, no constipation, no stomach pain."<br><br>To choose 'Very severe': She would be constipated that week. She would have more reflux, a lot more stomach pain."	

		reflux, no constipation, no stomach pain."<br><br>To choose 'Very severe': She would be constipated that week. She would have more reflux, a lot more stomach pain."<br><br>"Participant 4:<br>"Just how often we've had problems with just GI issues, feeding tube issues, feeding overall."<br>"Participant 4:<br>"I think it would probably be mildly severe for us just given all the issues we're having with venting. And I mean, other than that, she's keeping her feeds down really well. We have a lot of gas problems right now."<br><br>To choose 'Very severe': "We struggle with a lot of GI issues with her, it's so miserable. It's so frustrating. Because it's hard to troubleshoot. It's really hard. So that's the big one for us."<br><br>To choose ' No symptoms': "That would be amazing. I don't think I've had that in the past. In the past 13 months."<br><br>"Participant 5:<br>"Well, it's asking me to rate how her stomach symptoms have been and feeding has been	"Participant 4:<br>"Just how often we've had problems with just GI issues, feeding tube issues, feeding overall."<br><br>"Participant 4:<br>"I think it would probably be mildly severe for us just given all the issues we're having with venting. And I mean, other than that, she's keeping her feeds down really well. We have a lot of gas problems right now."<br><br>To choose 'Very severe': "We struggle with a lot of GI issues with her, it's so miserable. It's so frustrating. Because it's hard to troubleshoot. It's really hard. So that's the big one for us."<br>To choose ' No symptoms': "That would be amazing. I don't think I've had that in the past. In the past 13 months."<br><br>"Participant 5:<br>"Well, it's asking me to rate how her stomach symptoms have been and feeding has been in the past seven days, what I've kind of observed."<br><br>"Participant 5:<br>"It's kind of in between the severe and mildly severe." "Because she's had a lot of stomach issues, but then also ... See, again, non-verbal. I know I keep saying that. But she's been uncomfortable."<br><br>To choose 'Very Severe': "I would have hooked up the pump all these seven days."<br><br>To choose 'No Symptoms': "We would be	

		in the past seven days, what I've kind of observed."<br><br>"Participant 5:<br>"It's kind of in between the severe and mildly severe."<br>"Because she's had a lot of stomach issues, but then also ... See, again, non-verbal. I know I keep saying that. But she's been uncomfortable."<br><br>To choose 'Very Severe': "I would have hooked up the pump all these seven days."<br><br>To choose 'No Symptoms': "We would be running her feed at the normal rate the whole night, and I wouldn't have to stop the feed at all, not even one time. And I wouldn't have to vent her in the middle of the night like I've been doing. She would not be fussy. No gagging, no retching. Not a lot of secretions. I've been suctioning her. Not having to take the syringe and dump it in the cup and get it back in her with her meds that are coming out of her."	running her feed at the normal rate the whole night, and I wouldn't have to stop the feed at all, not even one time. And I wouldn't have to vent her in the middle of the night like I've been doing. She would not be fussy. No gagging, no retching. Not a lot of secretions. I've been suctioning her. Not having to take the syringe and dump it in the cup and get it back in her with her meds that are coming out of her."	
**Seizures and Myoclonic Jerks**	The level of severity regarding my child's seizures (or myoclonic jerks) over the past 7	"Participant 1:<br>"How bad have the seizures been in the last 7 days? It's numbers, it's length of time, how severe the seizure is, how involved it is. Those are all	"Participant 1:<br>"How bad have the seizures been in the last 7 days? It's numbers, it's length of time, how severe the seizure is, how involved it is. Those are all indicators of how bad."	

	days has been	indicators of how bad."<br><br>"Participant 1:<br>"He does have the seizures where his whole body jerks. That's our bad seizure, that's like a severe seizure for us. The little, like, startle seizures he has, they concern me, but they're just quick, a few seconds and they're over with. But I look at the number on those, if he's having a lot of those, something's wrong."<br><br>"Participant 2:<br>N/A"<br><br>"Participant 2:<br>"Mildly. Okay, we're going with mildly."<br><br>To choose 'Very Severe': "Oh yeah, two weeks ago she had to actually stay an hour and a half later, because he had an hour-long seizure."<br><br>"Participant 3:<br>"How, how many seizures she's had this week"<br><br>"Participant 3:<br>"Mildly, severe." Because she had the one instance that was severe, and yeah."<br><br>To choose 'No symptoms': "I'm probably not having very much	 "Participant 1:<br>"He does have the seizures where his whole body jerks. That's our bad seizure, that's like a severe seizure for us. The little, like, startle seizures he has, they concern me, but they're just quick, a few seconds and they're over with. But I look at the number on those, if he's having a lot of those, something's wrong."<br><br>"Participant 2:<br>N/A"<br><br>"Participant 2:<br>"Mildly. Okay, we're going with mildly."<br><br>To choose 'Very Severe': "Oh yeah, two weeks ago she had to actually stay an hour and a half later, because he had an hour long seizure."<br><br>"Participant 3:<br>"How, how many seizures she's had this week"<br><br>"Participant 3:<br>"Mildly, severe." Because she had the one instance that was severe, and yeah."<br><br>To choose 'No symptoms': "I'm probably not having very much seizure activity to where it affects her, like puts her to sleep, or makes you cry or anything like that."<br><br>To choose 'Severe': "Very strong seizures that make her more tired, sleep for longer periods of time, and then the crying seizures."	

		seizure activity to where it affects her, like puts her to sleep, or makes you cry or anything like that."<br><br>To choose 'Severe': "Very strong seizures that make her more tired, sleep for longer periods of time, and then the crying seizures."<br><br>"Participant 4:<br>"Just the level of severity that I'm dealing with overall of the seizures or myoclonic jerks that she had."<br><br>"Participant 4:<br>N/A"<br><br>"Participant 5:<br>"The question means what I saw or observed with the myoclonic jerks or seizures. The past seven days, what I've observed and what the severity level was."<br><br>"Participant 5:<br>"So I'd say mildly severe." "I think often the medicine's helped out."<br><br>To choose 'Very severe': "Very severe would be ... again, it would warrant a call to the doctor or having her at the hospital."	"Participant 4:<br>"Just the level of severity that I'm dealing with overall of the seizures or myoclonic jerks that she had."<br><br>"Participant 4:<br>N/A"<br><br>"Participant 5:<br>"The question means what I saw or observed with the myoclonic jerks or seizures. The past seven days, what I've observed and what the severity level was."<br><br>"Participant 5:<br>"So I'd say mildly severe. I think often the medicine's helped out."<br><br>To choose 'Very severe': "Very severe would be ... again, it would warrant a call to the doctor or having her at the hospital."<br><br>To choose 'No symptoms': "That would be a perfect world. That would be she didn't have RCDP. She might have something else, but she didn't have RCDP."	

		To choose 'No symptoms':<br>"That would be a perfect world. That would be she didn't have RCDP. She might have something else, but she didn't have RCDP."		
**Respiratory Symptoms**	The level of severity regarding my child's respiratory symptoms over the past 7 days has been	"Participant 1:<br>"Has my child had concerning respiratory issues over the last 7 days. Like has he needed an increase in oxygen, is he coughing more, is he working harder to breathe, are we suctioning him more?"<br><br>"Participant 1:<br>'No Symptoms.'<br><br>To choose 'Very severe': "When he's getting sick and it's a respiratory sickness."<br><br>"Participant 2:<br>N/A"<br><br>"Participant 2:<br>"I'd say mild, probably. Still not really back to his baseline that I would call his normal baseline."<br><br>"Participant 3:<br>"Just, has she had any issues, respiratory-wise, this week?"<br><br>"Participant 3:<br>"maybe not severe." "She just had a little bit of coughing and	"Participant 1:<br>"Has my child had concerning respiratory issues over the last 7 days. Like has he needed an increase in oxygen, is he coughing more, is he working harder to breathe, are we suctioning him more?"<br><br>"Participant 1:<br>'No Symptoms.'<br><br>To choose 'Very severe': "When he's getting sick and it's a respiratory sickness."<br><br>"Participant 2:<br>N/A"<br><br>"Participant 2:<br>"I'd say mild, probably. Still not really back to his baseline that I would call his normal baseline."<br><br>"Participant 3:<br>"Just, has she had any issues, respiratory-wise, this week?"<br><br>"Participant 3:<br>"maybe not severe." "She just had a little bit of coughing and just... More for reflux issues, but it... She's gagging a bit."<br><br>To choose ' Very severe': "A lot of	

		just... More for reflux issues, but it... She's gagging a bit."<br><br>To choose ' Very severe': "A lot of coughing and gagging, and worry that she's taking stuff in and not been able to clear it."<br><br>"Participant 4:<br>"Overall, how is your child's respiratory health?"<br><br>"Participant 4:<br>"No symptoms." "So she really has not had any symptoms over the past week. We've been really lucky."<br><br>To choose 'Very severe':<br>"Anytime you're talking about respiratory issues, if your child's having a lot of trouble breathing, it's really scary. It's an indicator of pretty serious health problems and bad."<br><br>"Participant 5:<br>"This question is asking me what I've observed or noticed in the past seven days for her respiratory issues, with her breathing and pulmonary issue wise, what the severity of it, if there's been any symptoms or anything showing that she's junky and stuff."<br><br>"Participant 5:	coughing and gagging, and worry that she's taking stuff in and not been able to clear it."<br><br>"Participant 4:<br>"Overall, how is your child's respiratory health?"<br><br>"Participant 4:<br>"No symptoms." "So she really has not had any symptoms over the past week. We've been really lucky."<br><br>To choose 'Very severe': "Anytime you're talking about respiratory issues, if your child's having a lot of trouble breathing, it's really scary. It's an indicator of pretty serious health problems and bad."<br><br>"Participant 5:<br>"This question is asking me what I've observed or noticed in the past seven days for her respiratory issues, with her breathing and pulmonary issue wise, what the severity of it, if there's been any symptoms or anything showing that she's junky and stuff."<br><br>"Participant 5:<br>"I think she's gotten better with the medication that we've been doing, I would say. We're definitely suctioning her a lot, so it's between not severe and mildly severe."<br><br>To choose 'Very Severe': "We'd either be doing extra treatments or we would be in the hospital with her."	

		"I think she's gotten better with the medication that we've been doing, I would say. We're definitely suctioning her a lot, so it's between not severe and mildly severe."<br><br>To choose 'Very Severe': "We'd either be doing extra treatments or we would be in the hospital with her."<br><br>To choose 'No Symptoms': "That never happens. She's always got some issues respiratory-wise. That's why we have to do so many treatments a day."	To choose 'No Symptoms': "That never happens. She's always got some issues respiratory-wise. That's why we have to do so many treatments a day."	
**Treatment and Therapy**	My child has been bothered by treatment or medical interventions	Participant 1:<br>"Did any therapies or any medications bother him?"<br><br>Participant 2:<br>"N/A"<br><br>Participant 3:<br>"Has she had any issues with medication, or anything like that, over the past seven days?"<br><br>Participant 4:<br>"I guess overall, obviously how much is the treatment, how many side effects or what's the treatment doing to your child?"<br><br>Participant 5:<br>"It's asking me by my	Participant 1:<br>"Not at All' for us over the last 7 days."<br><br>To choose 'Very Much': "If he's sick and he's on antibiotics or medicine that make him sick, that would be when I choose 'Quite a Bit' or 'Very Much.'"<br><br>Participant 2:<br>"Not at all."<br><br>Participant 3:<br>"Not at all."<br><br>To choose 'Very Much': "She's reacting to a new med that we've just given her, or she's sick and she's needing to go to the hospital."<br><br>Participant 4:	

		observation to rate the side effects of the treatments and therapy, for the medicine, if it's bothered her in the past seven days, what I've given her. The medication."	"Not at all. We're really, really lucky. my child's in really good health right now. And she's only taking two medications, I guess she takes reflux medication and she takes a daily prophylactic antibiotic."<br><br>To choose 'Very much': "It's horrible. If you feel like what you're doing to try to treat whatever symptom your child has is causing them additional discomfort or issues. I would feel like her quality of life would be not very good."<br><br>Participant 5:<br>"I would say somewhat. Because the one medicine she was on was causing her, they think, to have more seizures, so we had to get her off it."<br><br>To choose 'Very Much': "That she'd be on a lot more different medications that we're not used to. That we don't know what the side effects, how she reacts to the different medication, like we do all of her typical medicines that she's on now."<br><br>To choose 'Not at all': "That we were just on the normal medicines and we wouldn't have to worry about all the different... She's got a lot of side effects to all her medicine, so I don't know how to answer that, to be honest with you."	
