# Supplementary material for: Development and evaluation of RhizoQOL, a quality-of-life caregiver-reported survey for rhizomelic chondrodysplasia punctata, a rare peroxisomal disorder
Source: Orphanet J Rare Dis. 2025 Mar 31;20:147. doi: 10.1186/s13023-025-03660-0 (PMC11956500; doi:10.1186/s13023-025-03660-0)
Supplement: Supplementary file 3 — Supplementary Material 3: Supplementary Table 3: Required magnitude of treatment effect when response scores less than 2.0 are omitted. Hypothetical post-treatment scores based on longitudinal data response scores that are 2.0 or above [file 13023_2025_3660_MOESM3_ESM.pdf]

Supplementary Table 3: Required magnitude of treatment effect when response scores less than 2.0 are omitted

Question Item	Mean	Score Required for Significant Treatment Effect	Required Magnitude of Treatment Effect
**All Question Items**	**2.94**	**2.35**	**0.59**
Psychosocial Domain - Item 1	2.50	1.91	0.59
Psychosocial Domain - Item 2	2.16	1.91	0.25
Psychosocial Domain - CGI	Insufficient Data	Insufficient Data	Insufficient Data
Mobility Domain - Item 1	3.01	2.67	0.34
Mobility Domain - Item 2	2.64	2.39	0.25
Mobility Domain - Item 3	2.30	2.03	0.27
Mobility Domain - CGI	2.33	2.06	0.26
Feeding/GI Domain - Item 1	2.90	2.29	0.61
Feeding/ GI Domain - Item 2	2.92	2.23	0.69
Feeding/GI Domain - Item 3	3.29	3.02	0.27
Feeding/GI Domain - Item 4	2.77	2.02	0.75
Feeding/GI Domain - Item 5	3.59	1.99	1.59
Feeding/GI Domain - Item 6	3.50	2.91	0.59
Feeding/GI Domain - CGI	2.48	2.08	0.40
Seizures Domain - Item 1	3.54	3.12	0.42
Seizures Domain - Item 2	3.09	2.68	0.41
Seizures Domain - Item 3	Insufficient Data	Insufficient Data	Insufficient Data
Seizures Domain - Item 4	3.08	2.42	0.65
Seizures Domain - Item 5	3.06	1.95	1.10
Seizures Domain - Item 6	3.12	2.48	0.64
Seizures Domain - CGI	2.52	2.20	0.31
Respiratory Domain - Item 1	3.48	3.01	0.46
Respiratory Domain - Item 2	Insufficient Data	Insufficient Data	Insufficient Data
Respiratory Domain - Item 3	3.64	2.63	1.00
Respiratory Domain - Item 4	4.00	2.55	1.45
Respiratory Domain - Item 5	Insufficient Data	Insufficient Data	Insufficient Data
Respiratory Domain - CGI	2.49	1.91	0.58
Treatments Domain - Item 1	2.85	2.44	0.41
Treatments Domain - Item 2	2.65	2.03	0.62
Treatments Domain - Item 3	Insufficient Data	Insufficient Data	Insufficient Data
Treatments Domain - CGI	2.40	2.05	0.35
